# Structure-Based Discovery of Novel Chemical Classes of Autotaxin Inhibitors

**DOI:** 10.3390/ijms21197002

**Published:** 2020-09-23

**Authors:** Christiana Magkrioti, Eleanna Kaffe, Elli-Anna Stylianaki, Camelia Sidahmet, Georgia Melagraki, Antreas Afantitis, Alexios N. Matralis, Vassilis Aidinis

**Affiliations:** 1 Institute for Bioinnovation, Biomedical Sciences Research Center Alexander Fleming, Fleming 34, 16672 Athens, Greece; Magkrioti@Fleming.gr (C.M.); eleanna.kaffe@yale.edu (E.K.); stylianaki@fleming.gr (E.-A.S.); cameliasidahmet@gmail.com (C.S.); 2 NovaMechanics Ltd, Princess De Tyras 16, 1065 Nicosia, Cyprus; Melagraki@novamechanics.com

**Keywords:** atotaxin, small molecules, ATX inhibitors, in silico screening

## Abstract

Autotaxin (ATX) is a secreted glycoprotein, widely present in biological fluids, largely responsible for extracellular lysophosphatidic acid (LPA) production. LPA is a bioactive growth-factor-like lysophospholipid that exerts pleiotropic effects in almost all cell types, exerted through at least six G-protein-coupled receptors (LPAR1-6). Increased ATX expression has been detected in different chronic inflammatory diseases, while genetic or pharmacological studies have established ATX as a promising therapeutic target, exemplified by the ongoing phase III clinical trial for idiopathic pulmonary fibrosis. In this report, we employed an in silico drug discovery workflow, aiming at the identification of structurally novel series of ATX inhibitors that would be amenable to further optimization. Towards this end, a virtual screening protocol was applied involving the search into molecular databases for new small molecules potentially binding to ATX. The crystal structure of ATX in complex with a known inhibitor (HA-155) was used as a molecular model docking reference, yielding a priority list of 30 small molecule ATX inhibitors, validated by a well-established enzymatic assay of ATX activity. The two most potent, novel and structurally different compounds were further structurally optimized by deploying further in silico tools, resulting to the overall identification of six new ATX inhibitors that belong to distinct chemical classes than existing inhibitors, expanding the arsenal of chemical scaffolds and allowing further rational design.

## 1. Introduction

Autotaxin (ATX) is a secreted glycoprotein, widely present in biological fluids [1], where it catalyzes the hydrolysis of lysophosphatidylcholine (LPC) into lysophosphatidic acid (LPA) [2,3]. Although matricellular properties have been suggested for ATX, most of the reported effects of ATX are attributed to extracellular LPA production. LPA is a bioactive growth-factor-like lysophospholipid that exerts pleiotropic effects in almost all cell types, exerted through at least six G-protein-coupled receptors (LPAR1-6) [4,5] and leading to the stimulation of various cellular responses such as migration, proliferation and survival.

**Figure 1:**
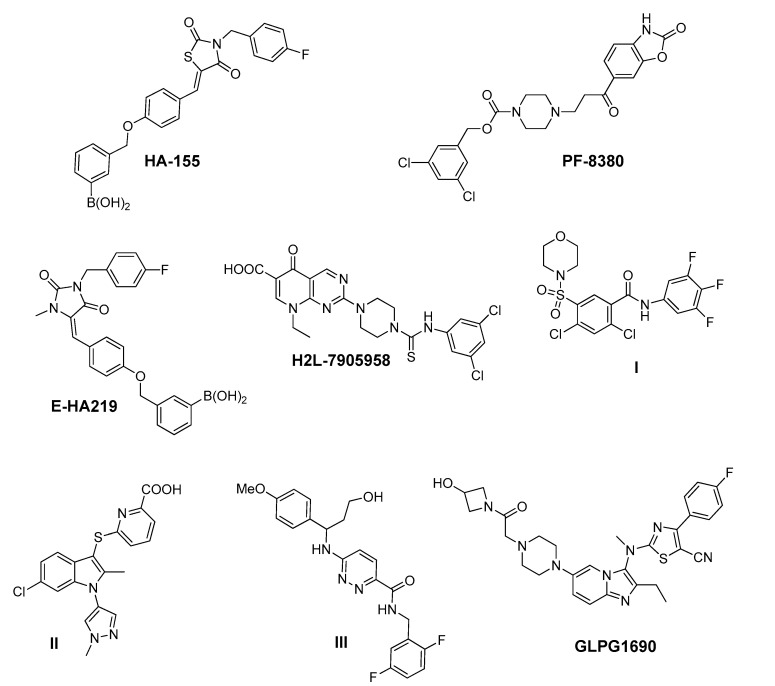
Representative chemical structures of known Autotaxin (ATX) inhibitors reported in the literature, and GLPG1690—the first-in-class ATX inhibitor—entered in advanced clinical trials in patients suffering from idiopathic pulmonary fibrosis.

ATX is vital for embryonic development, since ATX null mice suffer from serious vascular and neural defects, finally dying at embryonic day E9.5 [6]. In adult life, ATX is primarily expressed by adipose tissue, reproductive organs and the central nervous system [1] while inducible depletion of ~80% of ATX in adult mice did not cause any gross phenotypic defects, suggesting that the majority of ATX activity is dispensable for adult life and is a safe drug target [7]. However, increased ATX expression has been found in the arthritic synovium and serum of arthritic patients and animal models [8,9,10], in pulmonary fibrotic tissues of humans and animal models [11], in hepatic fibrotic tissues [12] and in patients of other chronic inflammatory diseases such as metabolic disorders [13], cholestatic disorders [14,15] and chronic hepatitis C [12,16,17,18]. Therefore, ATX has emerged as a major player in chronic inflammation [1,8,19], regulating processes such as vascular homeostasis, lymphocyte trafficking, platelet aggregation, stroma remodeling, metabolism, resistance to chemo- and radiotherapy and others [1,20]. In agreement, genetic deletion or pharmacological inhibition of ATX significantly attenuated modeled arthritis [10], bleomycin-induced lung fibrosis [11], hepatic fibrosis/hepatocellular carcinoma [12], obesity-induced hepatic steatosis [21] and resistance to chemo- or radiotherapy [22] in different experimental animal models, thus establishing ATX as a possible therapeutic target in chronic inflammatory diseases. Accordingly, the first-in-class ATX inhibitor GLPG1690 developed by Galapagos (Figure 1) [23] is currently in advanced clinical trials for the treatment of idiopathic pulmonary fibrosis patients (NCT03733444).

The crystal structure of ATX has been found to comprise two somatomedin B-like domains (SMB1 and SMB2) at its *N*-terminus stabilized by four pairs of disulfide bonds, a central catalytic phosphodiesterase (PDE) domain and a nuclease-like domain (NUC) at its *C-terminus* [5]. In particular, the catalytic domain of ATX consists of (a) a deep hydrophobic pocket that accommodates the substrate LPC, and (b) an active site containing a nucleophile Thr residue adjacent with two zinc ions, coordinated by conserved His and Asp residues [24,25]. Furthermore, ATX appears to have an allosteric hydrophobic channel which can accommodate the product LPA. This channel forms a T-junction with the active site and the hydrophobic pocket [5,26,27,28] and may serve as an entrance for the LPC substrate, and an exit for LPA, thus offering the necessary hydrophobic milieu for LPA delivery to its receptors [29]. 

The crucial role of ATX in the onset and progress of a multitude of severe disorders has attracted the interest of both the academic and industrial community towards the development of potent ATX inhibitors as drug-targets. Accordingly, several series of ATX inhibitors have been developed in the last decade (Figure 1) [27,30,31,32,33,34,35,36,37,38,39]. Some of them were discovered by performing high-throughput screening methods while others by rational design, using the solved crystal structure of ATX co-crystallized with inhibitors [24,25,40,41].

Among the ATX inhibitors reported, several metal chelators have been studied, such as the natural aminoacid l-Histidine, exhibiting an IC_50_ value in the millimolar range, as well as ethylenediamine-tetraacetic acid (EDTA) and 1,10-phenanthroline, displaying a better effect on ATX inhibition [27,42]. In addition, lipid and lipid-based ATX inhibitors, reminiscent of LPC and LPA structure, have been developed, including cyclic phosphatidic acid- (cPA) and α-bromomethylene phosphonate-like (BrP-LPA) derivatives [5], various thiophosphates [43,44,45], sphingosine analogues [46] and β-keto and β-substituted phosphonate chemotypes based on a tyrosine building block (VPC8a202) [47,48]. The most potent, thus far, lipid-based inhibitor, S32826, has been identified by a high-throughput screening process of 13,000 diverse compounds on ATX activity and exhibits an IC_50_ value of 5.6 nM in the LPC assay [49].

In addition to substrate-based inhibitors, some representative series of small molecule ATX inhibitors have also been reported, among which the boronic acid derivative HA-155, its bioisostere E-HA219 [50], as well as the piperazine analogue PF8380 developed by Pfizer [51] (Figure 1). All these derivatives were found to be among the most powerful ATX inhibitors (both in vitro and ex vivo in human whole blood) reported to date in the literature. Recently, ONO Pharmaceutical introduced the tetrahydrocarboline-based inhibitor ONO-8430506, with IC_50_ values of 5.1 nM and 4.5 nM in the FS-3 and LPC assay, respectively [52,53]. Other ATX inhibitors that have been developed include the pipemidic acid-based molecule H2L-7905958 (Figure 1) [54,55], various antioxidants, such as polyphenols and phenolic acids [56], benzene-sulfonamide-based derivatives (I, Figure 1) [31,57], indole-thioether carboxylic acid derivatives (II, Figure 1) (Inc. 2012), pyridazines (III, Figure 1) and tetrahydropyridopyrimidine derivatives [58,59], as well as benzoxazolone or benzotriazole- [60], imidazole- [61] and benzonaphthyridinamine-based analogues [62,63].

Despite the recent progress in developing ATX inhibitors by both the academic and the industrial sector, only a handful of them are endowed with a considerable drug-like profile, with the other ones suffering either from poor drug-like properties or lack of a clear mechanism of action [27]. Notably, from a therapeutic standpoint, the recent entry and the so far very promising results of the first-in-class ATX inhibitor GLPG1690 (Figure 1) [23] in advanced clinical trials against idiopathic pulmonary fibrosis lends support to the viability and validity of this approach, bringing it to the forefront of drug discovery efforts [23,27]. This drug candidate is currently being evaluated in advanced phase III clinical trials (NCT03733444) [64], having already exhibited a favorable safety profile and pharmacological effect in phases I and II trials, respectively [65,66]. 

Consequently, inhibition of ATX constitutes a tractable approach and the development of ATX small molecule inhibitors is considered a novel strategy to combat severe human diseases. In accordance with the aforementioned, the present study describes a multidisciplinary approach addressing to the application of cheminformatics tools accompanied by in vitro assays, aiming at identifying structurally novel ATX inhibitor hits covering a broad spectrum of chemical diversity. These hits could be helpful structural starting points towards developing potent ATX inhibitors endowed with optimal biopharmaceutical properties by using straightforward medicinal chemistry approaches. Therefore, a number of more than 14,000 pure diverse small molecules included in the HitFinder database (Maybridge) were virtually screened by a structure-based framework. Based on these results, a prioritized list of 30 compounds was created that was further tested in vitro to assess compounds’ inhibitory activity against ATX, with a well-established enzymatic assay (Amplex Red). From the experimental results received, two derivatives with weak ATX potency (KM03601 and SCR01013, Table 1) emerged. The latter analogues were further optimized by using computational tools (see Experimental part), through searching similar compounds in the PubChem database. Finally, six ATX inhibitor hits with potency in the low micromolar range were identified (Table 1) representing novel chemical classes of ATX inhibitors, well-amenable to further medicinal chemistry modifications to improve potency, pharmacokinetics/pharmacodynamics (PK/PD) profile and absorption, distribution, metabolism, and excretion–toxicity (ADMET) properties.

## 2. Results and Discussion

**Scheme 1:**
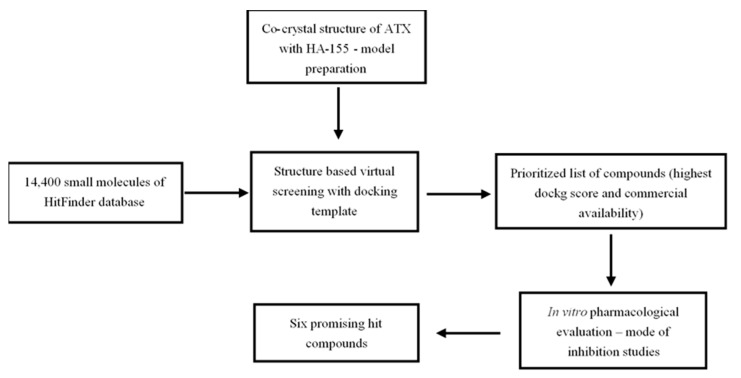
Workflow followed for the identification of the six most promising small molecule ATX inhibitor hits described in the context of the present study.

Mindful of Autotaxin’s (ATX) pivotal role in the pathogenesis of chronic inflammatory and fibroproliferative diseases, the aim of the present study was the development of an efficient in silico drug discovery workflow, with the purpose of identifying potent and structurally novel ATX inhibitors (Scheme 1). For this reason, the crystal structure of ATX (ENPP2) (PDB code: 2XRG) in complex with the boronic acid derivative inhibitor HA-155 (Figure 1) [25] served as a molecular model for our investigation, while MolegroVirtual Docker 5.0 was used as a docking software. 

The co-crystallized ligand HA-155 was used as template for the evaluation of the accuracy of the MolegroVirtual Docker 5.0 to produce the initial binding mode of the crystal structure (PDB code: 2XRG) with a 0.98 Å root mean square deviation. The docking pose of HA-155 with ATX showed that the mode of binding of HA-155 in ATX (Figure S1) is in accordance with previously developed crystallography studies pointing out that this compound is engaged in interactions with Thr210 residue, while the 4-fluorobenzyl moiety reaches deep in the hydrophobic pocket of the enzyme which accommodates both the substrate (LPC) and product (LPA) lipid chains. It is also suggested that the boronic acid group interacts not only with the threonine oxygen nucleophile, but also with the two zinc ions that are essential for the catalytic activity of ATX. The thiazolidine-2,4-dione core-ring of HA-155 and the conjugated aromatic ring are located between the hydrophobic pocket and the catalytic site. Furthermore, the ether linker, connecting the benzylboronic acid and the benzylidene moiety in HA-155, is well exposed to solvent. No hydrogen bonds or salt bridges seem to get involved in HA-155 binding to ATX (Figure S1). 

In addition, we also conducted docking studies with another known and well-studied ATX inhibitor developed by Pfizer, PF8380 (Figure 1). The docking pose (Figure S1) suggested that the lipophilic tail of this molecule (3,5-dichlorobenzylcarbamate) occupies the hydrophobic pocket, mimicking the binding mode of LPC substrate, the acidic headgroup (benzo[d]oxazol-2(3H)-one) interacts with the two zinc cations of the catalytic site, while the core spacer (piperazine) provides PF8380 with an appropriate conformation so that the lipophilic and the acidic groups interacting in an optimal fashion with the hydrophobic and the catalytic site of ATX, respectively.

**Figure 2:**
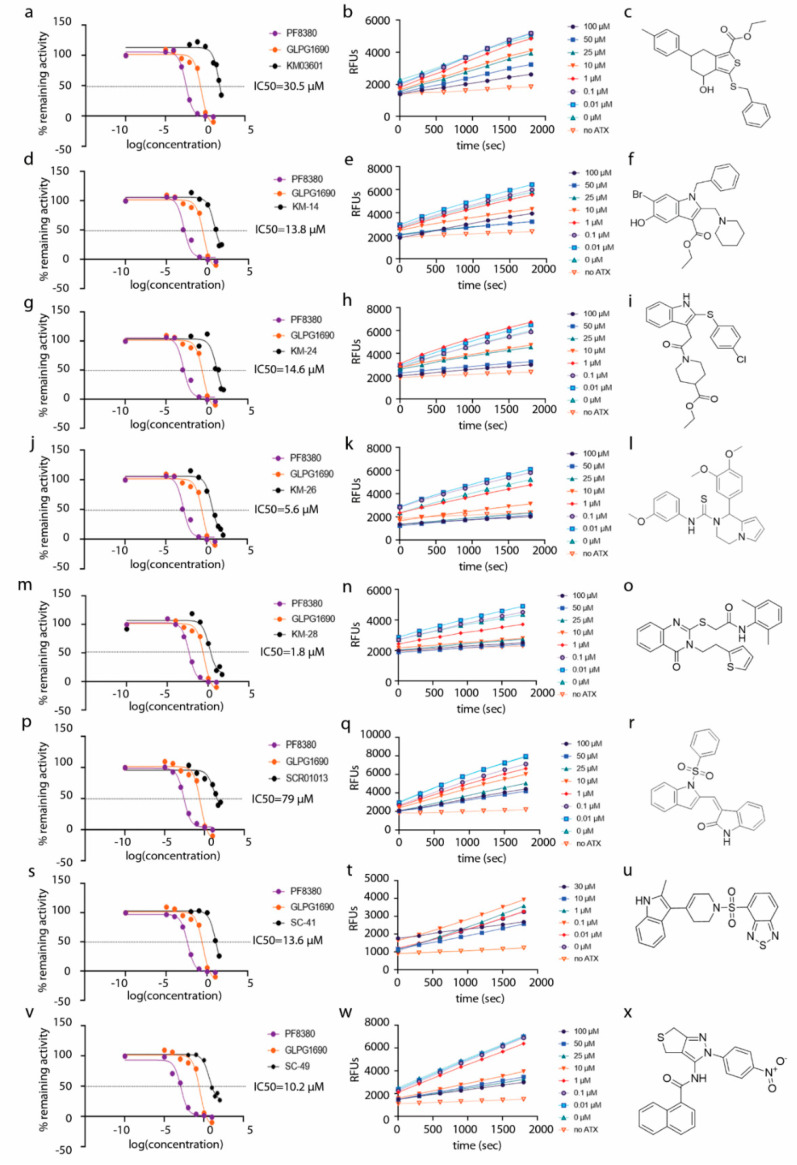
Dose–response inhibition graphs (**a**,**d**,**g**,**j**,**m**,**p**,**s**,**v**), kinetic graphs (**b**,**e**,**h**,**k**,**n**,**q**,**t**,**w**) and chemical structures (**c**,**f**,**i**,**l**,**o**,**r**,**u**,**x**) of human ATX inhibitors identified in this study. Reference compounds PF-8380 and GLPG-1690 are also included in the dose–response graphs.

The whole spectrum of 14,400 compounds included in the HitFinder database (Maybridge) was selected based on conforming to Lipinski’s “rule-of-five” for drug-likeness, and subsequently docked into the enzyme active site. The final binding modes of the compounds, after performing the respective docking runs, were selected based on the highest score (best fit), which corresponds to the complex with the lowest free energy of binding (ΔGb). For the docking poses depicted in Figure S1, the conformations with the lowest ΔGb of the compounds were used. Based on the structure-based investigation and conformation visual inspection, we ended up with a proposed prioritized list of 30 compounds (Table S1) with a wide spectrum of molecular diversity, which was further pharmacologically evaluated in vitro (Scheme 1), with the Amplex Red Assay. The code names and the in vitro results of all compounds, accompanied by the results of HA-155, PF-8380 and GLPG-1690 which were used as reference compounds, are summarized in Table S1. Among these agents, two analogues (KM03601, SCR01013, Table 1) exhibited a weak inhibitory activity against human ATX (IC_50_ values of 30.5 and 79.0 µΜ, respectively, Table 1, Figure 2 and Figure S1), while all the other derivatives were found to be inactive (IC_50_ > 100 µΜ, Table S1). Accordingly, the lowest-energy docked poses of the new identified derivatives SCR01013 and KM03601 (Figure S1) showed favorable interactions with ATX. 

In a subsequent attempt to develop more potent ATX inhibitors, the two derivatives identified from the first round, SCR01013 and KM03601 (Table 1), were structurally optimized by deploying further in silico tools and exploiting extensively the PubChem database [68]. A virtual screening procedure was initially used for the identification of the first 1000 “neighbors” of the aforementioned analogues in the chemical space in terms of chemical similarity with the aid of Enalos Suite of tools [69] and Molecular Quantum Numbers (MQNs). Secondly, a priority list of potentially new inhibitors was constructed by applying a structure-based virtual screening model. The commercial availability of the new compounds was also checked and as a result 49 new compounds were finally processed to pharmacological evaluation in vitro: 26 compounds with a similarity to SCR01013 and 23 compounds to KM03601; the results are included in Table S2. Interestingly, after testing the new analogues in vitro (Figure 2), this optimization procedure led to the identification of six new ATX inhibitors: KM-14, KM-24, KM-26, KM-28, SC-41 and SC-49 (Table 1 and Table S2, Figure 2). Among these, KM-28 exhibited the most potent activity with an IC_50_ value of 1.8 µΜ (Table 1, Figure 2). The IC_50_ values of the other derivatives are also included in Table 1. In addition, all the compounds of this study obey to Lipinski’s rule of five since they have no more than one violation of the specific Lipinski’s criteria (Table S3).

All six compounds were also tested in terms of their inhibitory activity against mouse ATX, exhibiting more or less similar, compared to human ATX, inhibition (Table 1). What is more, because ATX belongs to the ectonucleotidepyrophosphatase/phosphodiesterase (ENPP) family, the three most active derivatives (KM-26, KM-28 and SC-49) of this study were also evaluated for their activity in the phosphodiesterase (PDE) assay. This colorimetric assay kit uses 3′,5′-cAMP and 3′,5′-cGMP as substrates, and it is useful for the screening of inhibitors of nucleotide phosphodiesterase activity. As displayed in Table 1, all three analogues tested exhibited an outstanding activity against PDE, with IC_50_ values being comparable to the respective values against human and mouse ATX.

**Figure 3:**
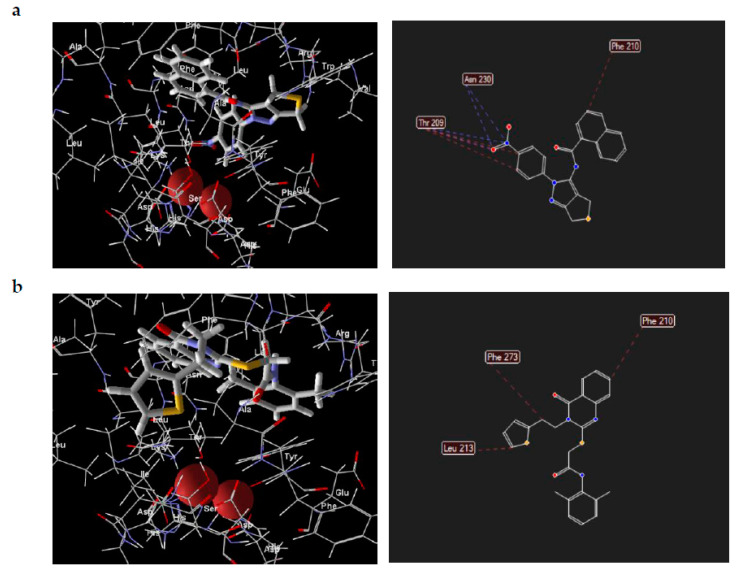
Docking poses and interactions of SC-49 (**a**) and KM-28 (**b**) in ATX.

Visual inspection of the lowest binding free energies of ATX complexes with two of our best inhibitors, KM-28 and SC-49, revealed different modes of binding (Figure 3). More specifically, the dihydrothienopyrazole core of SC-49 was found to occupy a pivotal spot in the center between the active site and the hydrophobic pocket of ATX, orienting the naphthyl and the 4-nitrophenyl substituents towards the lipophilic and the hydrophilic site, respectively (Figure 3). On the other hand, KM-28 seems to adopt a different conformation. Specifically, (a) the quinazolinone central core stands on top of the hydrophobic pocket of the catalytic site, with the thiophenyl ethyl side chain reaching deep in the same pocket developing hydrophobic interactions with aminoacids existing around, while (b) the 2,6-dimethylphenylsulfanyl acetamide moiety points in the direction of the allosteric hydrophobic channel (Figure 3).

The next step of our research focused on whether the ATX inhibitors depicted in Table 1 potentially interfere with the second or third reaction of our assay (Figure S2). As stated in the Experimental part, the in vitro assay employed (Amplex Red Lysophospholipase D assay) is the most common assay used for testing ATX inhibitors and consists of three reactions, namely (i) the hydrolysis of LPC to LPA and choline by ATX, (ii) the oxidation of choline by choline oxidase with the simultaneous production of H_2_O_2_, which is followed by (iii) the conversion of Amplex substrate by horseradish peroxidase (HRP), in the presence of H_2_O_2_, to the fluorescent resorufin. Therefore, a potential involvement of the new inhibitors with the second or third reaction of the assay would imply inhibition of the enzymes choline oxidase and/or HRP and subsequently misinterpreted results in regard to the real inhibition of ATX induced by the inhibitors. According to the graphs displayed in Figure S2, none of the inhibitors were found to inhibit either the second or third step of the assay, with the exception of KM-26 which showed a partial inhibition of the second/third reactions at high concentrations, but not at concentrations close to its IC_50_ value.

**Figure 4:**
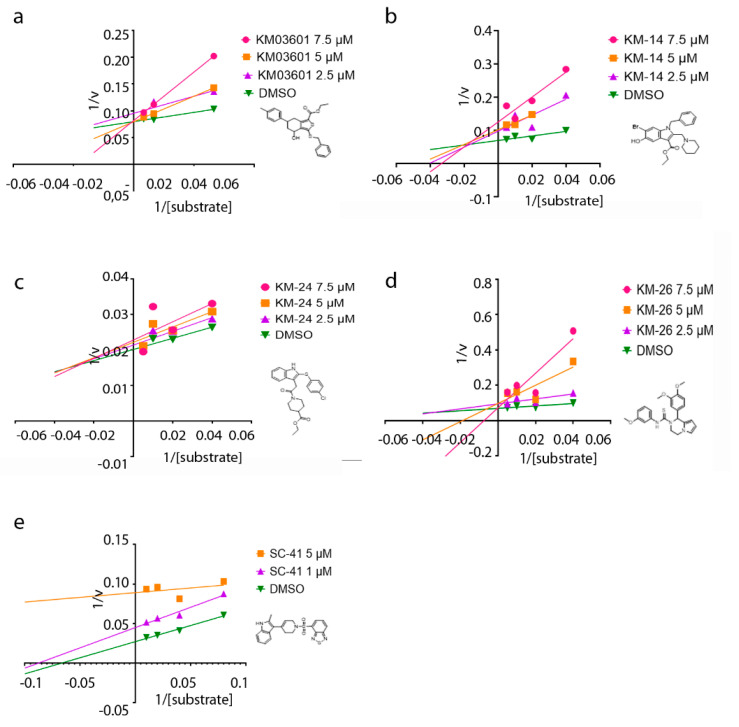
Mode of inhibition studies. Lineweaver–Burk plots displaying the reciprocal of enzyme activity against the reciprocal of substrate (LPC) concentration for compounds KM03601 (**a**), KM-14 (**b**), KM-24 (**c**), KM-26 (**d**) and SC-41 (**e**).

Lastly, to get insight into the mode of inhibition of the new ATX inhibitors developed in the present study, the Lineweaver–Burk plots were performed. The graphs of ATX enzyme activity in the presence of various concentrations of substrate (LPC) and inhibitors are depicted in Figure 4. It can be inferred from the graphs that the apparent Km values increase linearly with increasing concentrations of the inhibitors KM03601 and KM-26, without apparent change in Vmax, consistent with a competitive inhibition mechanism (Figure 4). As a result, both KM03601 and KM-26 bind only to free enzymes, and this binding occurs in the active site of ATX in the area where the substrate (LPC) also binds. Nevertheless, different behavior was observed for compounds KM-14 and KM-24, for which it was found out that they inhibit ATX in a mixed manner (Figure 4), meaning that these inhibitors can bind to ATX irrespectively of the substrate binding to the enzyme. Intriguingly, increasing concentrations of SC-41 was shown to decrease both the apparent Vmax and Km values (Figure 4), compatible with an uncompetitive mode of inhibition. Therefore, SC-41 seems to bind exclusively to the ATX-LPC complex affording finally an inactive enzyme-substrate-inhibitor complex. 

## 3. Materials and Methods 

### 3.1. Database

The HitFinder database (Maybridge) was selected to serve as the main source of available small molecules for initiating our workflow to the identification of ATX inhibitors. This database is comprised of 14,400 premier compounds representing the drug-like diversity of the Maybridge Screening Collection, thus offering easy and quick hit or lead identification. All compounds included conform to Lipinski’s rule-of-five for drug-likeness, and have purity greater than 90%. After evaluating several small molecule databases with millions of analogues each, a specific library was generated consisting of non-reactive compounds, thus ensuring fewer false positives and higher quality results, and subsequently offering the possibility of identifying new leads.

### 3.2. Virtual Screening

The crystal structure of ATX (ENPP2) (PDB code: 2XRG) in complex with the boronic acid derivative HA-155 (Figure 1) was used as a molecular model for our investigation. All compounds included in the HitFinder database were docked into the enzyme active site. The computational molecular docking studies were performed by using the MolegroVirtual Docker 5.0 software package (MVD version-5.0), while all structures were prepared using Molegro’s Molecules and Protein Preparation Wizard. Proper bond assignments, bond orders, hybridization and charges were also calculated by MVD. Explicit hydrogens were added and their hydrogen bonding patterns were determined by MVD as well. The MolDock Score (Grid), which is a grid-based scoring function pre-calculating potential-energy values on an evenly spaced cubic grid, was used for speeding up the calculations. A grid resolution of 0.30 Å was set to initiate molecular docking process and the binding site on the protein was defined based on the extension in all directions around the cavity (X, Y and Z) and with a radius of 15 Å. In order to generate the poses, a maximum of 1500 iterations was used combined with a population size of 50 using the default settings. Several diverse torsion angles, translations and rotations were generated to evaluate the affected part of the molecule with the aim to select the lowest energy conformation. The energy of the conformation is selected (according to ‘simplex evolution algorithm) only if it is below 100.0 which is the threshold in the particular study. The particular algorithm can execute local/global search of the generated poses. The simplex evolution algorithm was set to 300 maximum iterations, while the neighbor distance factor was set to 1.0. Neighbor distance factor is the factor determining how close the point of the initial simplex will be to the other randomly selected individuals in the population.

The 14,400 pure diverse small molecules from HitFinder database (Maybridge) were virtually screened on a structure-based framework and the highest scoring compounds from the virtual screening procedure were tested in vitro to assess their inhibitory activity against ATX. Our strategy for identifying these novel small molecule ATX inhibitors is briefly outlined in Scheme 1.

### 3.3. Optimization of the Initial Hits

The early identified by the initial in silico virtual screening approach and experimentally evaluated in vitro ATX inhibitors SCR01013 and KM03601 (Table 1, Figure 2), were subsequently used to optimize their structures using further computational tools. Towards this end, PubChem database was used for refining new structures. PubChem is the largest molecular database available in public (50 million entries), that archives the molecular structures and bioassay data within the National Institute of Health (NIH) Roadmap for Medical Research Initiative. This database was searched on the basis of 42 integer value descriptors of molecular structure, called Molecular Quantum Numbers (MQNs), enabling an understanding of the diversity and a useful visualization of this database spanning from small drug-like fragments to large natural products, polypeptides and oligonucleotides. MQNs can encode significant features of organic molecules, such as atoms, bonds, polar groups and topological features. A space is generated (MQN-space) which classifies molecules according to global structural features and biological activity. These distances can assist in the identification of known drugs but also can be used to measure the similarity between pairs of molecules (form scalar fingerprint) in a ligand-based virtual screening concept.

### 3.4. Pharmacological Evaluation

All compounds proposed were assessed for their ATX inhibitory activity in vitro using the Amplex Red assay. The Amplex Red Lyso-phospholipase D assay is a fluorometry-based assay providing a sensitive method for measuring Lyso-phospholipase D (ATX) activity in vitro. In this enzyme-coupled assay, ATX activity is monitored indirectly using 10-acetyl-3,7-dihydrophenoxazine (Amplex Red reagent), a sensitive fluorogenic probe for H_2_O_2_. ATX initially cleaves the Lysophosphatidylcholine (LPC) substrate to yield choline and Lysophosphatidic acid (LPA), while choline gets subsequently oxidized by choline oxidase to betaine and H_2_O_2_. Lastly, H_2_O_2_, in the presence of horseradish peroxidase (HRP), reacts with Amplex Red reagent in a 1:1 stoichiometry to generate the highly fluorescent product resorufin. 

The inhibition assay was performed as previously described [70] with some modifications in order to accommodate the newly-discovered lag phase in ATX kinetics [71]. To this end, and in order to provide adequate time to the hydrolysis of LPC by ATX, we added a 30min incubation period between ATX, inhibitors and LPC. Briefly, 2 μL of different concentrations of inhibitors diluted in DMSO (Sigma-Aldrich, St. Louis, MO, USA) were incubated with 50 µL of ATX 8 nM (human or mouse ATX from Sino Biological), and 48 µL reaction buffer (50 mM Tris-Cl pH 8.0 from Thermo Fisher Scientific, Waltham, MA, USA and 5 mM CaCl_2_ from Honeywell-Fluka, Charlotte, NC, USA) in a black 96-well microplate for 15 min at 37 °C. Thence, another 50 µL of reaction buffer with 200 μM LPC substrate 16:0 (Avanti Polar Lipids Inc, Alabaster, AL, USA) (final concentrations of LPC and ATX are 50 µM and 2 nM, respectively) was added to the reaction mixture and incubated for 30 min at 37 °C in order for ATX to convert LPC to LPA and choline. Finally, 50 µL of working solution containing 200 μM Amplex Red (Invitrogen, Carlsbad, CA, USA), 4 U/mL HRP (Sigma-Aldrich, St. Louis, MO, USA), 0.4 U/mL choline oxidase (Sigma-Aldrich, St. Louis, MO, USA) in 50 mM Tris-HCl pH 8.0 and 5 mM CaCl_2_ was added to measure the produced choline. The reaction was monitored at 37 °C in a fluorescence plate reader using excitation at 530 nm and reading at 590 nm, every 5 min for 30 min. The velocity of the reaction and the remaining activity of ATX were calculated at the linear phase of the reaction and dose-response inhibition graphs were plotted with GraphPad Software (GraphPad Software, San Diego, CA, USA). IC_50_ value was determined as the inhibitor concentration that results in a 50% inhibition of the enzyme activity. In order to evaluate a possible effect of the compounds on the second (choline oxidase) and third (HRP) enzymes of the Amplex Red assay, we also performed the assay in the absence of ATX but in the presence of the compounds. Instead of ATX, we added choline chloride at a final concentration of 2 µM so as the second and third steps of the assay could proceed normally.

To determine the mode of inhibition of ATX by each inhibitor, a modified Amplex assay was employed. In brief, various concentrations of inhibitors (depending on their IC_50_ values: 2.5, 5 and 7.5 µΜ for KM-14, KM-24, KM-26 and KM03601 and 1 and 5 µM for SC-41) were tested against various substrate (LPC) concentrations (25, 50, 100 and 200 µΜ for KM-14, KM-24, KM-26, KM03601 and 12.5, 25, 50, 100 μM for SC-41). Mouse ATX from Sino Biological was used in all mode of inhibition assays. Thereafter, the velocity (V) of the reaction was calculated and the reciprocal of velocity (1/V) was plotted against the reciprocal of substrate concentration (1/S) in Lineweaver–Burk graphs using GraphPad Software (GraphPad Software, San Diego, CA, USA).

## 4. Conclusions

In this article, the identification of novel and structurally diverse ATX inhibitor hits with potency in the low micromolar range is described, through performing an efficient two-step structure-based in silico drug discovery workflow followed by in vitro enzymatic assays. Despite the inherent limitations, in silico structure-based virtual screening was shown to be proficient in the identification of novel ATX inhibitors. The increasing availability of crystal structures of ATX with different inhibitors will allow further optimization of existing leads as well as the discovery of new ones.

Moreover, the newly identified compounds belong to distinct chemical classes than existing inhibitors, expanding the arsenal of chemical scaffolds for further rational design. The ensuing lead optimization will be further assisted by molecular dynamics, while the analysis of the ADMET properties of the most potent compounds will facilitate prioritization.

## Figures and Tables

**Table 1:** In vitro inhibitory activity against human and mouse ATX for the most promising compounds identified, using recombinant ATX at a final concentration of 2 nM and lysophosphatidylcholine (LPC) 16:0 as a substrate. The mode of inhibition is also mentioned.

Compound	Pubchem CID	Name	Structure	IC_50_ (μΜ) [hATX/mATX]	Inhibition of 2nd/3rd Reaction	IC_50_ (μΜ) PDE Assay	Mode of Inhibition
KM03601	2820206	ethyl 3-benzylsulfanyl-4-hydroxy-6-(4-methylphenyl)-4,5,6,7-tetrahydro-2-benzothiophene-1-carboxylate	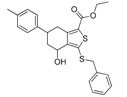	30.5/14.9	no	n.d.	competitive
KM-14	1201991	ethyl 1-benzyl-6-bromo-5-hydroxy-2-(piperidin-1-ylmethyl)-1H-indole-3-carboxylate	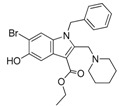	13.8/5.9	no	n.d.	mixed
KM-24	46379015	ethyl 1-[2-[2-(4-chlorophenyl)sulfanyl-1H-indol-3-yl]acetyl]piperidine-4-carboxylate	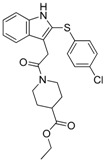	14.6/6.5	no	n.d.	mixed
KM-26	4662990	1-(3,4-dimethoxyphenyl)-*N*-(3-methoxyphenyl)-3,4-dihydro-1H-pyrrolo[1,2-a]pyrazine-2-carbothioamide	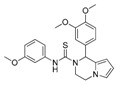	5.6/7.4	yes	2.8	competitive
KM-28	46925664	*N*-(2,6-dimethylphenyl)-2-[4-oxo-3-(2-thiophen-2-ylethyl)quinazolin-2-yl]sulfanylacetamide	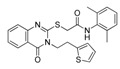	1.8/3.0	no	0.9	n.d.
SCR01013	5702991	(3Z)-3-[[1-(Benzenesulfonyl)indol-2-yl]methylidene]-1H-indol-2-one	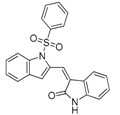	79.0/67.0	no	n.d.	n.d.
SC-41	20867222	4-{[4-(2-Methyl-1H-indol-3-yl)-3,6-dihydropyridin-1(2H)-yl]sulfonyl}-2,1,3-benzothiadiazole	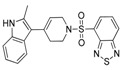	13.6/4.0	no	n.d.	uncompetitive
SC-49	3540718	*N*-[2-(4-nitrophenyl)-4,6-dihydrothieno[3,4-c]pyrazol-3-yl]naphthalene-1-carboxamide	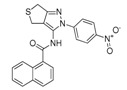	10.2/9.7	no	73.0	n.d.
HA155	46856189	[4-[[4-[(*Z*)-[3-[(4-fluorophenyl)methyl]-2,4-dioxo-1,3-thiazolidin-5-ylidene]methyl]phenoxy]methyl]phenyl]boronic acid	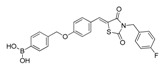	0.0025 (reported: 0.0057 [67])/n.d.	n.d.	n.d.	competitive
PF-8380	25265312	3,5-Dichlorobenzyl 4-(3-oxo-3-(2-oxo-2,3-dihydrobenzo[d]oxazol-6-yl)propyl)piperazine-1-carboxylate	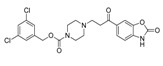	0.0025 (reported: 0.0017 [51])/n.d.	no	0.0011	competitive
GLPG1690	90420193	2-[[2-ethyl-6-[4-[2-(3-hydroxyazetidin-1-yl)-2-oxoethyl]piperazin-1-yl]-8-methylimidazo[1,2-a]pyridin-3-yl]-methylamino]-4-(4-fluorophenyl)-1,3-thiazole-5-carbonitrile	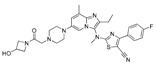	0.229 (reported: 0.131 [23])/n.d.	n.d.	n.d.	n.d.

## Supplementary Materials

Supplementary materials can be found at https://www.mdpi.com/1422-0067/21/19/7002/s1. Figure S1: Docking poses; Figure S2: Kinetics for the second and third reaction of the Amplex Red assay; Table S1: Results of the in vitro evaluation of the 30 compounds described in this study; Table S2: Results of the in vitro evaluation of the 49 new compounds derived from the second round of the in silico structural optimization study.

## Abbreviations

ADMET	Absorption, distribution, metabolism, excretion and toxicity
ATX	Autotaxin
Asp	Aspartic acid
ENPP2	Ectonucleotidepyrophosphatase/ phosphodiesterase
His	Histidine
LPC	Lysophosphatidylcholine
LPA	Lysophosphatidic acid
PK/PD	Pharmacokinetics/pharmacodynamics
Thr	Threonine
