# Supplementary material for: Structure-Based Discovery of Novel Chemical Classes of Autotaxin Inhibitors
Source: Int J Mol Sci. 2020 Sep 23;21(19):7002. doi: 10.3390/ijms21197002 (PMC7582705; doi:10.3390/ijms21197002)
Supplement: Supplementary file 1 [file ijms-21-07002-s001.zip › Supplementary Figures.pdf]

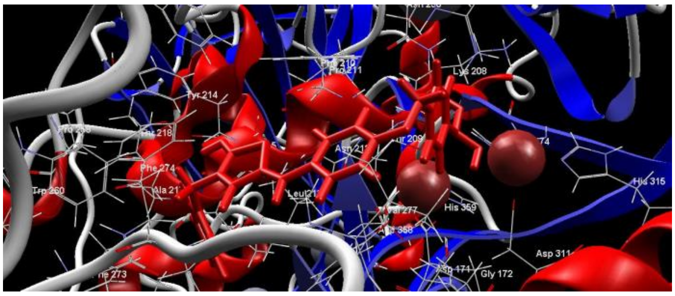

(a) **HA-155**

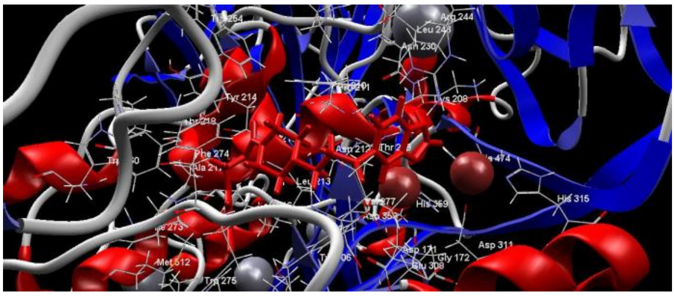

(b) **PF8380**

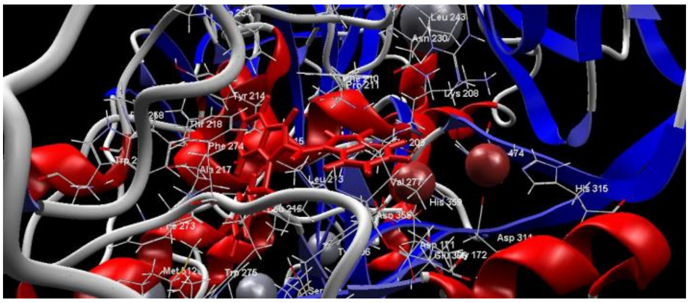

(c) **SCR01013**

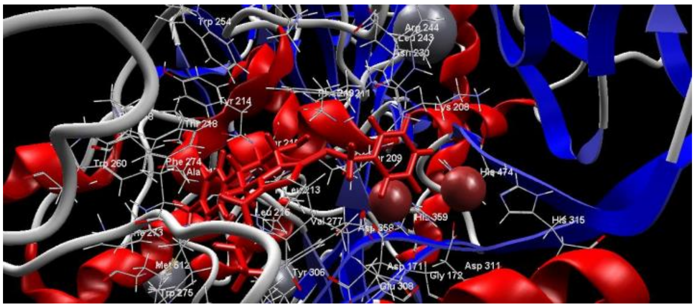

(d) **KM03601**

Figure S1. Docking poses illustrating the lowest-energy conformation of the reference compounds HA-155 and PF8380 (Fig. 1), as well as the initial hits SCR01013 and KM03601 (Table 1) bound to ATX.

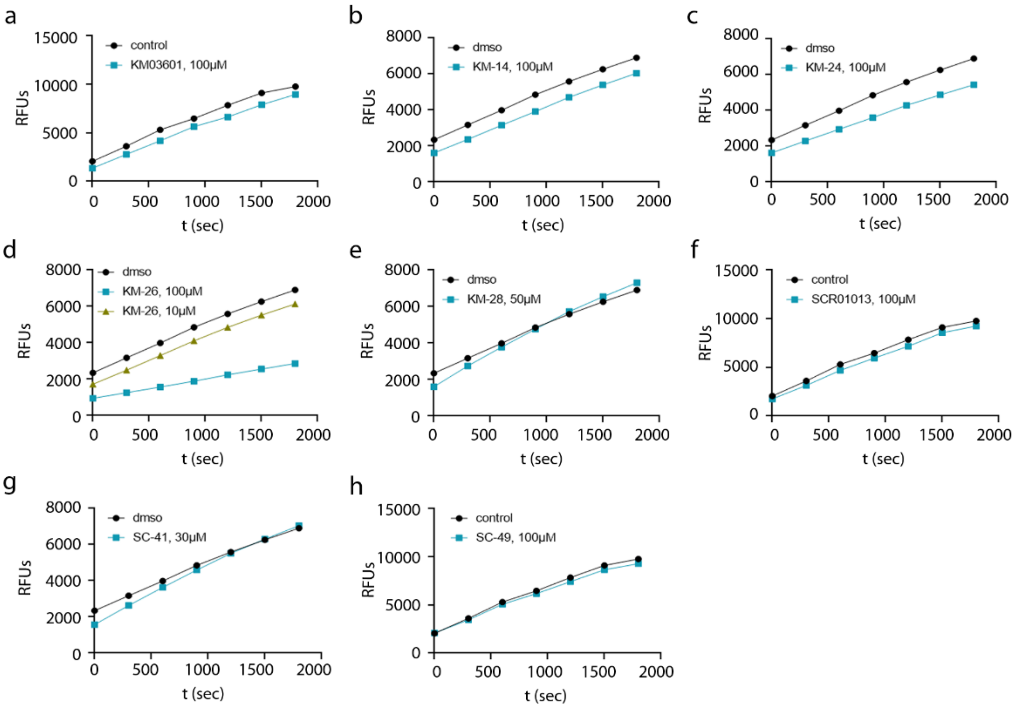

Figure S2. Kinetics for the second and third reaction of the Amplex Red Lyso-phospholipase D Assay. All compounds were tested at a final concentration of 100 µM apart from KM-28 and SC-41 which were tested at 50 µM and 30 µM, respectively, due to a lower concentration of their initial stocks.
